# Supplementary figures and images for: ESR1 gene amplification and MAP3K mutations are selected during adjuvant endocrine therapies in relapsing Hormone Receptor-positive, HER2-negative breast cancer (HR+ HER2- BC)
Source: PLoS Genet. 2023 Jan 3;19(1):e1010563. doi: 10.1371/journal.pgen.1010563 (PMC9839248; doi:10.1371/journal.pgen.1010563)

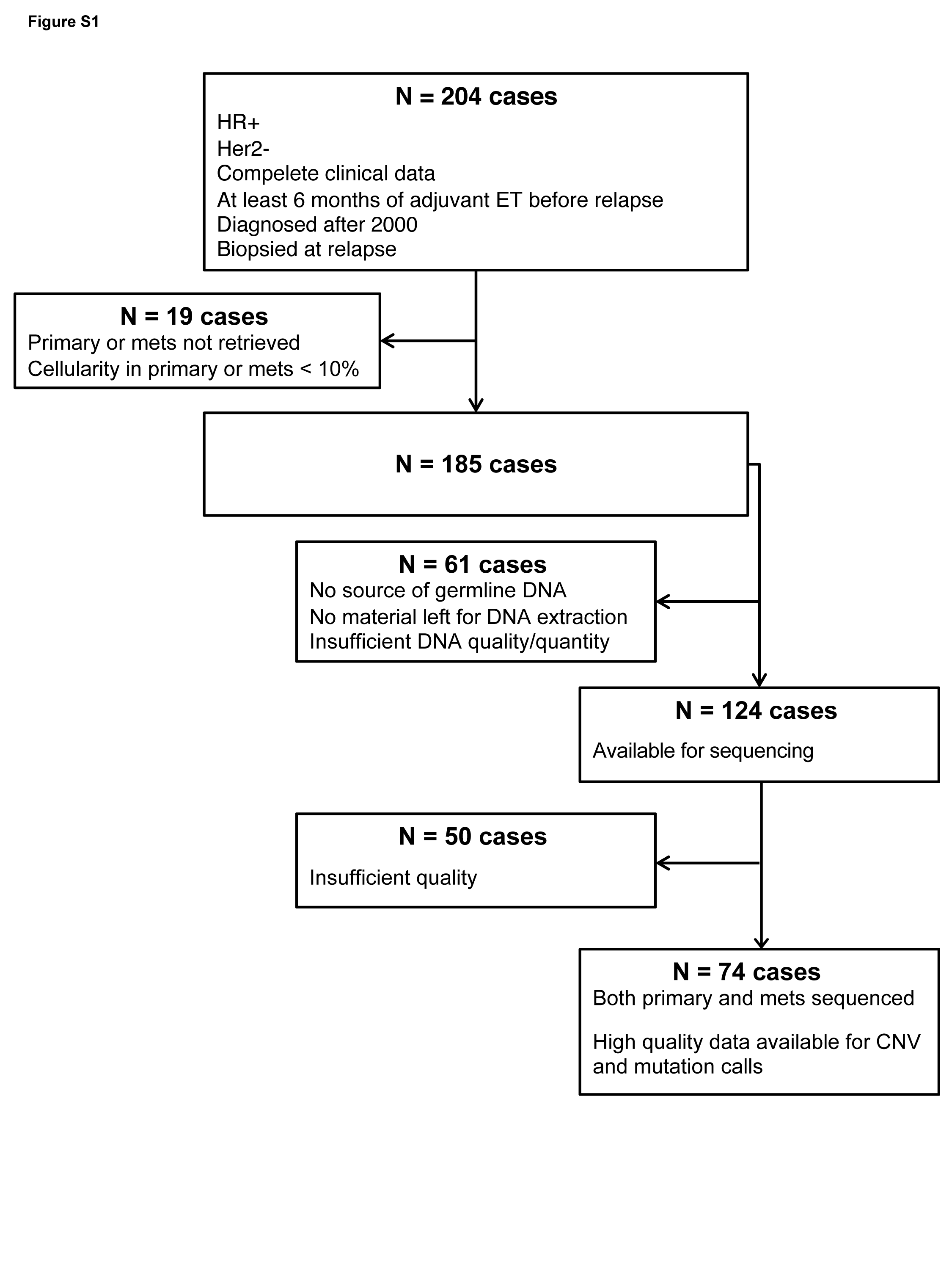

Supplement: S1 Fig — (PNG) [file pgen.1010563.s001.png]

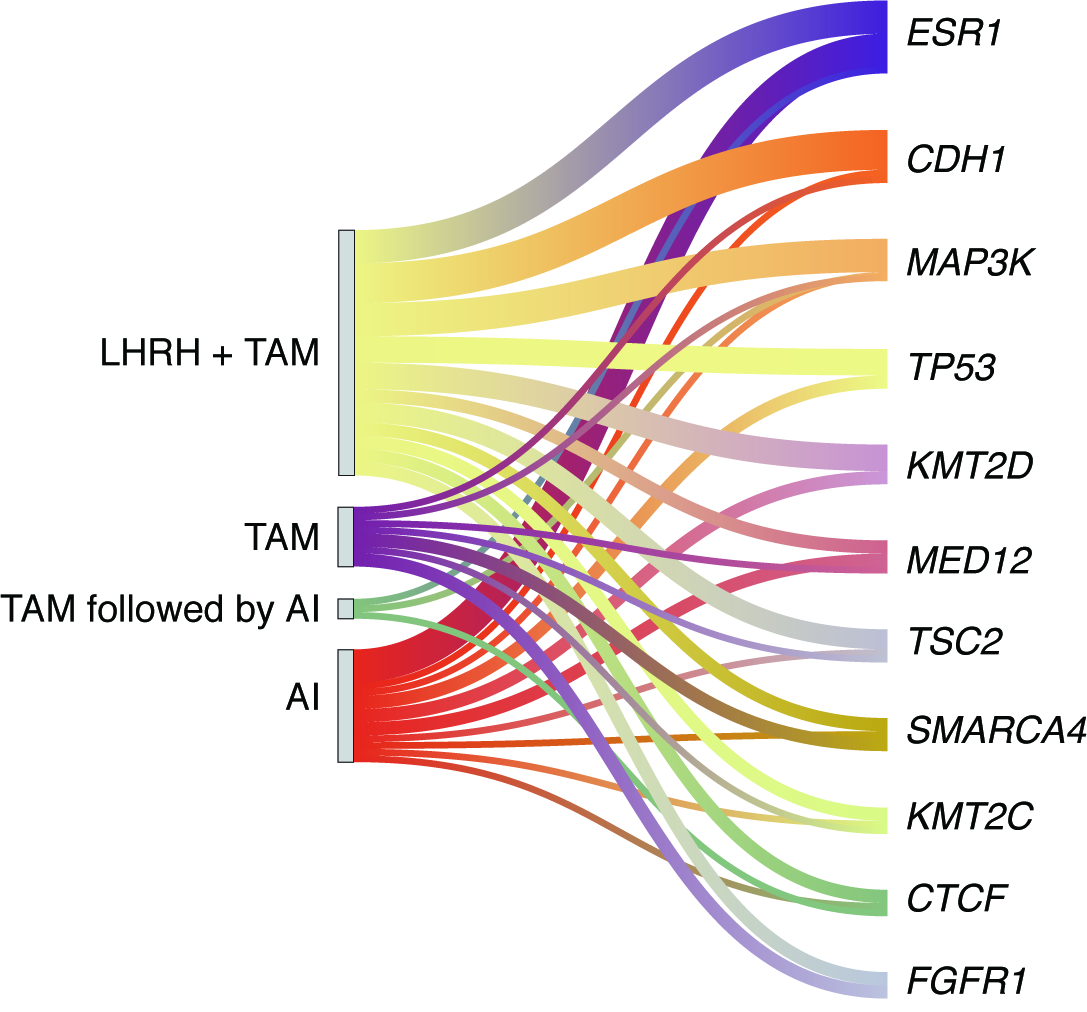

Supplement: S2 Fig — (TIF) [file pgen.1010563.s002.tif]
