# Supplementary material for: ESR1 gene amplification and MAP3K mutations are selected during adjuvant endocrine therapies in relapsing Hormone Receptor-positive, HER2-negative breast cancer (HR+ HER2- BC)
Source: PLoS Genet. 2023 Jan 3;19(1):e1010563. doi: 10.1371/journal.pgen.1010563 (PMC9839248; doi:10.1371/journal.pgen.1010563)
Supplement: S1 Star Methods — (DOCX) [file pgen.1010563.s008.docx]

**METHODS DETAILS**

**Pathology and *ESR1* amplification FISH assessment**

All primary and metastatic tumor samples from patients included in the study were retrieved from IEO pathology archives and reviewed by two expert pathologists (G.P., A.V.) for diagnosis confirmation. Tumor type, grade, ER, PgR and HER2 status, Ki-67 labeling index, occurrence of peritumoral vascular invasion, nodal status and type of surgery were recorded. Tumors were defined as HR+ HER2- BC on the basis of ASCO/CAP criteria (i.e., ≥1% of ER and/or ≥1 of PgR immunoreactivity and absence of HER2 overexpression or *HER2* gene amplification)^1^. ER/PgR and HER2 immunoreactivity was assessed according to the clinical procedures at the time of diagnosis^2-3^. In particular, mouse monoclonal antibodies 1D5 (for ER, 1:100 dilution) and 1A6 (for PgR, 1:800 dilution) (both from Dako, Glostrup, Denmark) were used between 2000 and 2007, and FDA-approved ERα/PR PharmDX kits (Dako) thereafter; the percentage of ERα-positive and PgR-positive invasive cancer cells was semi-quantitatively annotated. HER2 immunoreactivity was assessed using the anti-cErbB2 polyclonal antibody (Dako, 1:1000) between 2000 and 2005, and with the HercepTest^TM^ (Dako) thereafter: the percentage of immunoreactive cells, type and intensity of the staining were detailed. Ki-67 labeling index was assessed with the MIB-1 monoclonal antibody (Dako, 1:200) by counting at least 500 invasive cancer cells at the tumor edge, as recommended by the International Ki-67 in Breast Cancer Working Group^4^. Somatic copy number alterations (sCNA) of *ESR1* genes were assessed by FISH, using Bacterial Artificial Chromosome (BAC) clones obtained from C.H.O.R.I. (bac-pac resources, Children’s Hospital Oakland Research Institute, California, US), labeled by means of nick translation (Nick Translation Reagent Kit, Abbott Molecular, Chicago, Illinois, US), and validated on normal metaphase spreads. FISH evaluations were performed on FFPE sections using standard protocols. Probe mixture employed for *ESR1* gene copy number included: 1) Spectrum Orange (Abbott Molecular, Des Plains, IL) *ESR1* (6q25.2) specific BAC clones (RP11-773O22, RP11-195J5, RP11-1105F15); 2) Spectrum Green (Abbott Molecular) labeled chromosome 6q control (*MYB* gene in 6q23.3) specific BACs (clones RP11-104D9, RP11-1105M6); 3) Spectrum Aqua labeled chromosome 6 centromeric probe (CEP6-D6Z1, Abbott Molecular). A minimum of 50 tumor cells were scored using a Leica DM 6000B (Wetzlar, Germany) microscope at 100x magnification with the appropriate fluorescence filters. The number of *ESR1* gene copies in each tumor cell was normalized to the copies of its centromere (CEP6, ratio 1 – R1) as well as to the copies of its neighbor gene *MYB* (ratio 2 – R2). Two levels of amplification were defined based on ratio 1 (R1) values: low-level *ESR1* amplification, as defined as R1 between 1.5 and <2, and high-level *ESR1* amplification, as defined as R1≥2. An R2 ≥2 was considered as evidence of focal *ESR1* gene amplification.

**Targeted gene sequencing panel design**

To cover the highest number of BC-related genomic aberrations, we designed a custom, amplicon-based, 6,812-amplicon targeted gene sequencing (TGS) genomic panel spanning 530.65 Kbps of the human exome. The panel was optimized by ThermoFisher Scientific for automated fluidic handling with two primer pools, using an Ion Chef^TM^ robot, and amplicons were designed to be used with FFPE degraded material. Targets were selected from publicly available BC-omics datasets at the time of TGS panel customization. In detail, sources for mBC sets were exome sequencing data from Lefebvre et al.^5^, targeted sequencing from the MSK-IMPACT^TM^ repository^6^, and from the AACR GENIE Consortium^7^ (last accessed Dec 27, 2017). Sources for eBC sets were the TCGA^8^ and the METABRIC^9^ datasets. Other resources were COSMIC^10^ (v83, Dec 7, 2017, <https://cancer.sanger.ac.uk/>), the Cancer Gene Census^11^ (last accessed Feb 20, 2018, <https://cancer.sanger.ac.uk/census>) and PharmKGB^12^ (last accessed Mar 5, 2018). Specific targets were included in the panel if their mutational frequency in either eBC or mBC was higher than 2%, if somatic copy number aberrations (sCNA) occurred in higher than 5% of eBC or mBC specimens, or if the difference in the occurrence of genetic alterations between mBC and eBC was higher than 2%. All BC genes reported in the Cancer Census for breast cancer were included in our panel. Regions containing polymorphisms associated with pharmacogenetic data and the term “breast cancer” from PharmKGB were also included. To reduce the risk of missing genes that are more rarely mutated in BC, we also resorted to use a) a “density criterion”, i.e., including genes with a mutational density (i.e., the number of reported mutations/Kbps of exonic length) greater than the 90^th^ percentile of other genes reported as mutated in the available databases (thus rescuing *BAP1*, *CDKN1B*, *CDKN2A*, *CHEK2*, *DNMT3A*, *EPHA3*, *FANCC*, *FOXA1*, *IKZF1*, *PIK3R1*, *RARA*), and b) an “isolated peak criterion”, i.e., including hotspot regions presenting with more than 10 mutations over a rolling window of 30 bps (*FGFR2*, *MED12*, *NUP93*, *SMAD4*). Finally, we included S*QLE*, whose expression was previously associated with BC cell resistance to endocrine therapies in our previous study^13^. The complete gene list as well as the BED file with the targeted regions are available as supplementary information (**S1 and S2 Tables**).

**DNA extraction, library preparation and sequencing**

After the identification of tumor areas on hematoxylin and eosin (H&E) slides, 10 of 5-µm thick unstained slides were cut from each archival FFPE sample and manually macrodissected to ensure the highest yield of nucleic acids from cancer cells. Tumor cellularity of each sample was recorded. DNA extraction, library preparation and sequencing were randomized to minimize the possibility of cross-contamination by processing samples from the same case in the same workday.

DNA from FFPE BC specimens and normal breast tissue was extracted using the Maxwell® RSC DNA FFPE Kit with a Maxwell® RSC Instrument (Promega Corporation, Madison, USA) according to the manufacturer’s instructions after incubation at 65°C overnight. The concentration and purity of DNA samples was measured using the Qubit™ dsDNA HS Assay Kit (Invitrogen™) on a Qubit™ 2.0 Fluorometer (Invitrogen™). Libraries were generated using 10 ng input DNA using ThermoFisher Scientific Ion Chef™ system according to the manufacturer’s instructions (20 amplification cycles and 16 min of annealing). Multiplexed libraries were then sequenced using a ThermoFisher Scientific Ion GeneStudio™ S5 Plus System by pooling seven cancer sample libraries and 24 germline libraries respectively, on Ion 550™ chips. TGS reads were aligned against the human genome reference hg38/GRCh38 using the Torrent Suite Software, Torrent TMAP aligner 5.12.27 with default parameter settings. BAM files were processed using the Ion Reporter™ Software version 5.12 on a dedicated Ion Reporter™ Server System. Filtering cutoffs for proceeding to downstream analysis were as follows: for files derived from normal tissues Q20 < 150,000,000 bases, number of mapped reads < 1,500,000, coverage depth < 100x, percent on target at 20x < 90%; for files originating from cancer samples Q20 < 700,000,000 bases, number of mapped reads < 7,000,000, coverage depth < 1000x, percent on target at 100x < 80%. Such values were chosen as the excess approximation of the 10th percentile of each considered measure for all the initially sequenced files.

**Mutation, copy calling, and variant prioritization**

Prior to mutation calling from BAM files, we created a sequence variant baseline (SVB) with a proprietary algorithm by ThermoFisher which resulted in a dataset-specific BED file based on 87 FFPE normal samples (as a source of germline DNA) that fulfilled the previously described QC criteria. The SVB detects and filters out sequencing errors deriving from FFPE artefacts, which could lead to false positive variant calls. Mutation calling was then performed using the default settings of the ThermoFisher somatic-germline paired mutation pipeline. To increase our confidence in the mutation calls, we independently ran a calling pipeline that included conversion to binary, sorting and indexing performed using sambamba^14^ version 0.7.0. Post-processing and variant identification were performed using the Genome Analysis Toolkit^15-16^ (GATK) version 4.1.3.0 best practices: adding read groups using picardTools^17^ 2.20.6 (http://sourceforge.net/projects/picard/files/picard-tools/), base quality recalibration using GATK BaseRecalibrator and GATK ApplyBQSR algorithms, and somatic variant calling using Mutect2​ against normal pre-processed bam files using the population the population germline resource af-only-gnomad.hg38.vcf.gz from the GATK resource bundle, with parameter *af-of-alleles-not-in-resource* = 0.001 and disabling *MateOnSameContigOrNoMappedMateReadFilter* filter. ​FFPE artefacts were estimated using GATK LearnReadOrientationModel and variants were further filtered with GATK FilterMutectCalls using the *ob-priors* argument and the output generated from the previous step to filter out FFPE artefacts.

To obtain the *bona fide* driver mutations list we proceeded as follows: firstly, we intersected QC-passing calls from the two aforementioned callers, labelling the resulting mutations as ‘validated’. Then, we labeled mutations as ‘private’ when they were present only in one of the samples of each matched pair, and as ‘shared’ if they were present in both samples of a matched pair. We removed all mutations included in the UCSC Genome Browser Common SNPs database (v151). We then considered SNVs and indels separately for downstream filtering. SNVs that were classified as non-confident by the IonReporter™ Server System due to the low frequency in both tumor and matched normal samples were removed from further analyses. SNVs classified as validated or shared or present in one of the following ClinVar (r.d. 20181128) categories: risk factor, pathogenic, likely pathogenic, drug response with a variant allele frequency (VAF) > 0.1 were retained. Non-hotspot SNVs were removed from the analysis if they were not validated or private. Missense mutations were classified as deleterious if the IonReporter™ annotator labelled them as hotspot (Oncomine hotspot GRCh38 v5.12) or met the threshold of one of three available functional annotators (Polyphen^18^, SIFT^19^, FATHMM-MKL^20^) reported as deleterious by the respective creators (Polyphen ≥ 0.85, SIFT ≤ 0.05, FATHMM-MKL ≥ 0.70). All the other missense mutations which were classified as ‘functionally benign’ as well as synonymous mutations were removed. SNVs known in the literature to be heavily affected by deamination processes were further inspected; if a SNV belonging to this group was classified as private or not validated and showed a frequency < 0.15 was filtered out. We then performed a rescue step to recover lower frequency SNVs likely to be truly present at subclonal VAF: if a shared SNV was present in only one of the samples belonging to a given pair after the aforementioned filtering steps, it was recovered. The recovered mutations had to show an allelic frequency > 0.025. In contrast, if a shared mutation was classified as non-confident for low coverage in the normal sample in one of the samples of a given pair, it was removed from both samples. We followed similar rules for indels, but with stricter thresholds due to the higher likelihood of false positives generated by the Ion Torrent sequencing technology. In particular, indels affecting genes known to be heavily altered by the deamination process with a VAF frequency < 0.20 and not validated or labelled as private were removed. Indels with a FATHMM score < 0.7 were removed. Indels underwent the same previously described rescue process. A final visual inspection was performed using IGV to rule out potentially remaining artifacts.

sCNA were computed using ONCOCNV^21^, optimized for amplicon-based TGS data. The copy number baseline was created using the 74 normal samples passing QC criteria and with both primary and metastatic matched samples. Ploidy and absolute copy number for each sample were estimated using ABSOLUTE^22^ (v2.0). The ABSOLUTE output was visually, independently inspected by three blinded trained researchers (GZ, MD, and FR) and a consensus optimal solution was selected for each sample. Amplifications were then defined as 3.5-fold standard upper deviation from the estimated mean absolute copy number of a given sample, whereas deletions were defined as 2.5-fold standard deviation below the said mean. Gene Ontology (GO) term enrichment was performed fitting a generalized linear model. GO terms were annotated using AnnotationDbi^23^ R package.

**References**

1. Goldhirsch A, Winer EP, Coates AS, et al. Personalizing the treatment of women with early breast cancer: highlights of the St Gallen International Expert Consensus on the Primary Therapy of Early Breast Cancer 2013. Ann Oncol. 2013;2206-2223.
2. Hammond ME, Hayes DF, Wolff AC et al. American Society of Clinical Oncology/College of American Pathologists guideline recommendations for immunohistochemical testing of estrogen and progesterone receptors in breast cancer. J Clin Oncol. 2010; 2784-2795.
3. Wolff AC, Hammond ME, Schwartz JN, et al. American Society of Clinical Oncology/College of American Pathologists guideline recommendations for human epidermal growth factor receptor 2 testing in breast cancer. Arch Pathol Lab Med. 2007;18-43.
4. Dowsett M, Nielsen TO, A'Hern R, et al. Assessment of Ki67 in breast cancer: recommendations from the International Ki67 in Breast Cancer working group. J Natl Cancer Inst. 2011;1656-1664.
5. Lefebvre C, Bachelot T, Filleron T, et al. Mutational Profile of Metastatic Breast Cancers: A Retrospective Analysis. PLoS Med. 2016;e1002201.
6. Zehir A, Benayed R, Shah RH, et al. Mutational landscape of metastatic cancer revealed from prospective clinical sequencing of 10,000 patients. Nat Med. 2017; 703-713.
7. AACR Project GENIE Consortium. “AACR Project GENIE: Powering Precision Medicine through an International Consortium.” Cancer discovery. 2017; 818-831.
8. Hoadley KA, Yau C, Hinoue T, et al. Cell-of-Origin Patterns Dominate the Molecular Classification of 10,000 Tumors from 33 Types of Cancer. Cell. 2018;291-304.e6.
9. Pereira B, Chin SF, Rueda OM, et al. The somatic mutation profiles of 2,433 breast cancers refines their genomic and transcriptomic landscapes. Nat Commun. 2016;7:11479.
10. Tate JG, Bamford S, Jubb HC, et al. COSMIC: the Catalogue Of Somatic Mutations In Cancer. Nucleic Acids Res. 2019;D941-D947.
11. Futreal PA, Coin L, Marshall M, et al. A census of human cancer genes. Nat Rev Cancer. 2004;177-183.
12. Whirl-Carrillo M, McDonagh EM, Hebert JM, et al. Pharmacogenomics knowledge for personalized medicine. Clin Pharmacol Ther. 2012;414-417.
13. Brown DN, Caffa I, Cirmena G, et al. Squalene epoxidase is a bona fide oncogene by amplification with clinical relevance in breast cancer. Sci Rep. 2016;19435.
14. Tarasov A, Vilella AJ, Cuppen E, Nijman IJ, Prins P. Sambamba: fast processing of NGS alignment formats. Bioinformatics. 2015;2032-2034.
15. DePristo MA, Banks E, Poplin R, et al. A framework for variation discovery and genotyping using next-generation DNA sequencing data. Nat Genet. 2011;491-498.
16. McKenna A, Hanna M, Banks E, et al. The Genome Analysis Toolkit: a MapReduce framework for analyzing next-generation DNA sequencing data. Genome Res. 2010;1297-1303.
17. Wysoker, A., Tibbetts, K., & Fennell, T. (2011). picardTools 1.5.3.
18. Ramensky V, Bork P, Sunyaev S. Human non-synonymous SNPs: server and survey. Nucleic Acids Res. 2002;3894-3900.
19. Ng PC, Henikoff S. SIFT: Predicting amino acid changes that affect protein function. Nucleic Acids Res. 2003;3812-3814.
20. Shihab HA, Rogers MF, Gough J, et al. An integrative approach to predicting the functional effects of non-coding and coding sequence variation. Bioinformatics. 2015;1536-1543.
21. Boeva V, Popova T, Lienard M, et al. Multi-factor data normalization enables the detection of copy number aberrations in amplicon sequencing data. Bioinformatics. 2014;3443-3450.
22. Carter SL, Cibulskis K, Helman E, et al. Absolute quantification of somatic DNA alterations in human cancer. Nat Biotechnol. 2012;413-421.
23. Pagès H, Carlson M, Falcon S et al. AnnotationDbi: Annotation Database Interface. 2018. Bioconductor.
