# Supplementary material for: ESR1 gene amplification and MAP3K mutations are selected during adjuvant endocrine therapies in relapsing Hormone Receptor-positive, HER2-negative breast cancer (HR+ HER2- BC)
Source: PLoS Genet. 2023 Jan 3;19(1):e1010563. doi: 10.1371/journal.pgen.1010563 (PMC9839248; doi:10.1371/journal.pgen.1010563)
Supplement: Attachment — Submitted filename: Plos Genetics - rebuttal 11_22.docx [file pgen.1010563.s009.docx]

**Response to reviewers**

Please find below our point by point replies to Reviewers comments.

**Reviewer #1: The authors of this article performed a genomic characterization of 74 surgically resected HR+ HER2- BC patients relapsing during or at the completion of adjuvant ET. They compared tumor genomic alterations in matched mBC and eBC tumor specimens: along with previously reported genomic alterations, including PIK3CA, TP53, CDH1, GATA3 and ESR1 mutations/deletions, they found that ESR1 gene amplification (confirmed by FISH) and MAP3K mutations were enriched in metastatic lesions as compared to matched primary tumors of patients treated with either adjuvant aromatase inhibitors or LHRH analogs + tamoxifen. Patients with tumor bearing MAP3K mutations in metastatic lesions had significantly worse survival in multivariate analysis.**

**The manuscript is well written and study results are clearly reported and discussed.**

We thank the Reviewer for the positive comments about our work.

**Some comments/suggestions:**

**1. In the Methods section, the authors state that study patients were selected based on the availability of clinical data regarding “adjuvant ET (if any)”. If adjuvant ET was mandatory and all study patients received adjuvant ET, please eliminate “(if any)”.**

We thank the Reviewer for highlighting this inaccuracy. The manuscript has been modified accordingly (elimination of “if any”, page 5, line 138).

**2. Among patients characteristics, the authors should report how many patients relapsed during adjuvant ET and how many had disease relapse after the completion of adjuvant ET.**

We thank the Reviewer for this suggestion. We included such info in the supplementary table 1.

**3. The authors report that 12 patients were treated with AI, 3 with LHRH + AI, 5 with Tam followed by AI, 11 with Tam alone, 40 with LHRH + Tam, and 3 patients were treated in random clinical trials (total: 74). Furthermore, they write (page 8, line 1) that in 2 patients, they were unable to retrieve information about the type of ET prescribed. Please clarify to which patients they refer.**

As reported in table 1 (Demographic and clinical characteristics of the study cohort), 3 patients for whom we were not able to access clinical data were enrolled in prospective randomized clinical trials. We apologize for the inconsistency, the text in page 9 (line 250-251) has now been modified.

**4. Among acquired aberrations detected by NGS, only ESR1 mutations were found to be significantly enriched in mBC specimens after adjustment for multiple testing. If so, the authors should better clarify that the observed enrichment of MAP3K mutations in metastatic lesions was not statistically significant after multiple testing.**

We thank the Reviewer for the observation. We agree that MAP3K mutations taken individually do not reach statistical significance for enrichment after multiple testing correction. We have better clarified this point in the text (page 8, line 277-278).

However, we would like to highlight the fact that the relevant finding related to MAP3K mutation enrichment is not just this phenomenon per se (not reaching significance but - as for MAP3K13 - characterized by the complete absence in primary samples of mutations in such gene). Indeed, it is the outstanding statistically significant association with clinical outcome variables (DRFS and OS) of MAP3K mutations that we believe should be stressed throughout the manuscript.

**5. It would be interesting to know DRFS and OS of study patients by PIK3CA mutation status. PIK3CA and ESR1 mutation status should also be included as covariates in multivariable Cox analysis.**

We have now performed the Kaplan-Meier analysis of the PIK3CA status in eBC as requested. As shown in the picture below, DRFS and OS were numerically higher in PIK3CA mutated patients, although not reaching statistical significance, reasonably due to the low number of patients included. Overall, these data are in line with previously published larger case series, showing better outcomes in patients harboring PIK3CA mutations.

We also introduced PIK3CA status of eBC and acquired ESR1 as covariates of the multivariable Cox model. We report the results in the following tables. As shown, MAP3K status remains significant after adjustment for PIK3CA and ESR1 status. Even if the inclusion of such covariates is important in principle, we believe that the number of degrees of freedom is too high for a formal inclusion in the main manuscript in proportion to the sample size of our cohort. However, if the Reviewer believes that our new analyses add to our work from an exploratory point of view, we will be glad to include them in the supplementary materials of the revised manuscript as a table or as a figure.

Disease-relapse free survival:

| **Variable** | **HR** | **2.5 %** | **97.5 %** | **p-value** |
| --- | --- | --- | --- | --- |
| MAP3K status  Altered vs wild-type | 3.51 | 1.51 | 8.12 | 0.003 |
| ESR1 status  Altered vs wild-type | 0.82 | 0.39 | 1.70 | 0.60 |
| PIK3CA status  Altered vs wild-type | 0.83 | 0.49 | 1.43 | 0.51 |
| T size  pT2 or greater vs pT1 | 1.90 | 1.11 | 3.26 | 0.01 |
| N status  Positive vs Negative | 1.35 | 0.72 | 2.51 | 0.34 |
| Ki67  LumB-like vs LumA-like | 1.61 | 0.97 | 2.68 | 0.06 |
| Menopausal status  Post vs Pre | 1.41 | 0.80 | 2.45 | 0.23 |
| Grade  G3 vs G2, G1, Gx | 1.42 | 0.84 | 2.35 | 0.18 |

Overall survival:

| **Variable** | **HR** | **2.5 %** | **97.5 %** | **p-value** |
| --- | --- | --- | --- | --- |
| MAP3K status  Altered vs wild-type | 5.36 | 2.10 | 13.67 | 0.0004 |
| ESR1 status  Altered vs wild-type | 1.13 | 0.48 | 2.64 | 0.77 |
| PIK3CA status  Altered vs wild-type | 0.85 | 0.43 | 1.67 | 0.64 |
| T size  pT2 or greater vs pT1 | 1.59 | 0.82 | 3.08 | 0.16 |
| N status  Positive vs Negative | 1.85 | 0.79 | 4.33 | 0.15 |
| Ki67  LumB-like vs LumA-like | 2.34 | 1.18 | 4.61 | 0.01 |
| Menopausal status  Post vs Pre | 1.65 | 0.83 | 3.27 | 0.14 |
| Grade  G3 vs G2, G1, Gx | 2.69 | 1.38 | 5.25 | 0.003 |

**Reviewer #2: This study by Lorenzo et al. presents genomic analysis of ER+ breast tumors that recurred after endocrine therapy. Resistance to endocrine therapy is a significant problem in ER+ breast cancer and the question is important. Strengths of the study include matched primary tumor, germline DNA, and recurrent tissue for direct comparison of acquired features. This study additionally adds granular analysis of how patients were treated. Study is limited by relatively small sample size compared to prior works (Razavi) and lack of mechanistic or translational studies. Additionally, the two main alterations described, ESR1 and MAPK are well described.**

We thank the Reviewer for the positive comments, highlighting the strengths of our study and the significance of the clinical questions addressed by our manuscript. As clearly stated in the discussion session, one of the main limitations of our study is its relatively small sample size. However, the key aspect of our study is the presence of matched samples, whereas in Razavi et al., even though an high overall number of patients were included (n=1918 patients), only a small case subset was represented by matched primary/metastatic samples (n=74 patients).

Moreover, we would like to highlight that the NGS analysis presented in Razavi et al. were performed using the MSK-IMPACT® multicancer panel, which does not cover infrequently mutated but potentially relevant genes identified in breast cancer studies throughout the last 15 years. In our work, we devised, based on an extensive genomic database review, a custom-made panel specifically designed to cover known and potential breast cancer associated genes. The design of our panel is described in detail in the supplementary materials of our manuscript, and is a freeware tool we made available to the scientific community at large. Moreover, we would like to highlight that our ESR1 CNV findings are unique in the literature of breast cancer genomic studies, in that, for the first time to our knowledge, they were corroborated by transversal validation using FISH on all the dataset, thus increasing their analytical and biological validity.

**- Table 1 describes luminal A like and Luminal B-like based on Ki-67 - why was a more established transcriptomic definition not utilized?**

Patients included in the study were not originally classified into intrinsic molecular subtypes by transcriptomic analysis. As described in the methods section, we adopted the St. Gallen criteria for the clinicopathological surrogate definition of intrinsic subtypes, still representing the mainstay in breast cancer diagnosis and treatment. However, even if the distinction between Luminal-A and Luminal-B breast cancers is important for patients’ management, we did not perform further transcriptional analyses and decided to adopt a widely used and accepted clinico-pathological classification of HR+ breast cancer to select our cohort.

**- Figure 2 B is confusing to me. It seems that the treatment should be on the left because it occurs temporally before the alterations.**

We thank the Reviewer for the input. The figure is indeed confounding. We amended the figure and the relative capture as suggested.

**The two diagrams also present largely overlapping data and I would encourage the authors to pick one or clearly highlight the value of both.**

We agree with the Reviewers that the two figures present lots of overlapping elements. We moved the diagram detailing the expanded treatment in the supplementary material.

**- The ESR1 by treatment type sensitivity analysis is based on a very small number of patients and the strength of conclusions (ESR1 mutations and amplifications is driven by estrogen deprivation) should be toned down.**

We thank the reviewer for the suggestion, we toned down the message in the results session (line 350, page 13).

**- Another explanation is that rather than driving mutagenesis (which is possible), that low abundance subpopulations harbor mutations and then are selected by therapy. Thus mutations may confer differential sensitivity to treatment which then manifest as different growth rates under selective pressures. This alternative hypothesis should be discussed.**

We wish to thank the Reviewer for their precious input. Indeed, we believe their explanation to be extremely interesting. In line with the Reviewer’ observations, we presented a case harboring ESR1 mutation, in which the variant allele frequency raised from 2,6% in the primary tumor sample to 8,8% in the metastatic sample, speculating that this could represent an example of clone expansion upon therapy selective pressure. We took the liberty of paraphrasing their alternative explanation of our finding and expanding it as a part of our discussion (lines 474-481, page 19).
